# Biological Properties and Absolute Configuration of Flavanones From *Calceolaria* *thyrsiflora* Graham

**DOI:** 10.3389/fphar.2020.01125

**Published:** 2020-07-28

**Authors:** Ernesto Valdés, César González, Katy Díaz, Yesseny Vásquez-Martínez, Carolina Mascayano, Claudia Torrent, Francisco Cabezas, Sophia Mejias, Margarita Montoya, Marcelo Cortez-San Martín, Marcelo A. Muñoz, Pedro Joseph-Nathan, Mauricio Osorio, Lautaro Taborga

**Affiliations:** ^1^ Laboratorio de Productos Naturales, Departamento de Química, Universidad Técnica Federico Santa María, Valparaíso, Chile; ^2^ Programa Centro de Investigaciones Biomédicas Aplicadas, Facultad de Ciencias Médicas, Escuela de Medicina, Universidad de Santiago de Chile, Santiago, Chile; ^3^ Departamento de Biología, Facultad de Química y Biología, Universidad de Santiago de Chile, Santiago, Chile; ^4^ Departamento de Ciencias del Ambiente, Facultad de Química y Biología, Universidad de Santiago de Chile, Santiago, Chile; ^5^ Facultad de Ciencias, Instituto de Ciencias Químicas, Universidad Austral de Chile, Valdivia, Chile; ^6^ Departamento de Química, Centro de Investigación y de Estudios Avanzados del Instituto Politécnico Nacional, Mexico City, Mexico

**Keywords:** *Calceolaria thyrsiflora*, flavanone, absolute configuration, anti-inflammatory activity, antibacterial activity

## Abstract

Flavanones (–)-(2*S*)-5,4’-dihydroxy-7-methoxyflavanone (1) and (–)-(2*S*)-5,3’,4’-trihydroxy-7-methoxyflavanone (2) were isolated from the extracts of *Calceolaria* *thyrsiflora* Graham, an endemic perennial small shrub growing in the central zone of Chile. The absolute configuration of these compounds was resolved by optical rotation experiments and *in silico* calculations. Three analogs (3, 4, and 5) were synthesized to do structure-activity relationships with the biological assays studied. Biological tests revealed that only flavanone 2 exhibited a moderate inhibitory activity against the methicillin-resistant strain *S*. *aureus* MRSA 97-77 (MIC value of 50 µg/ml). In addition, flavanone 2 showed a potent, selective, and competitive inhibition of 5-hLOX, which supports the traditional use of this plant as an anti-inflammatory in diseases of the respiratory tract. Also, 2 exhibited cytotoxic and selective effects against B16-F10 (8.07 ± 1.61 µM) but 4.6- and 17-fold lesser activity than etoposide and taxol.

## Introduction

The South-American geography allows the growth of a great flora diversity with a high degree of endemism (Villagran and Hinojosa, 1997) where different *Calceolaria* species are found. This genus of the spermatophytes belongs to the Angiosperms division and is found from Patagonia to central Mexico, with most species growing near the Andes mountains (Molau, 1988). Thus, there are around 50 species in Chile, of which some have been used in the traditional Chilean medicine due to their antibacterial, diuretic, and digestive properties (Garbarino et al., 2007).

One of them, *C.* *thyrsiflora* Graham, that grows in steep, sunny, and eroded places, mainly in streams (Ministerio de Salud de Chile, 2010) blooms from September to December, is found between the provinces of Coquimbo and Curicó, both on coast and inland locations, and is also abundantly found in slopes of the surroundings of Santiago (Ehrhart, 2000). It is commonly known as “Herb or Sweet Stick” due to the intense dominant flavor of its leaves, whose infusion has healing effects and is popularly used to relieve sore throats, tonsils inflammation, and lesions of the oral mucosa (thrush and gum inflammation). Due to its astringent and diuretic properties, it is also used for the treatment of urinary incontinence and other renal or bladder conditions, as well as a sweetener to flavor drinks (Erazo Giuffra et al., 1987; Ministerio de Salud de Chile, 2010). Other studies on several *Calceolaria* species account for antimicrobial, antioxidant (Falcão et al., 2006), and insecticidal activities (Cespedes et al., 2016), while chemical studies allowed the characterization of aromatic, diterpene, triterpene, glycoside, and flavonoid secondary metabolites (Céspedes et al., 2013).

Herein we describe the antibacterial, antiproliferative, anti-inflammatory (inhibition of 5-hLOX), and antifungal effects of extracts of *C. thyrsiflora* and of the two isolated metabolites (–)-(2*S*)-5,4’-dihydroxy-7-methoxyflavanone (1) and (–)-(2*S*)-5,3’,4’-trihydroxy-7-methoxyflavanone (2), as well as the spectroscopic characterization and absolute configuration assessment of the two secondary metabolites by electronic and vibrational circular dichroism. In addition, the three analogous racemic flavanones **3**, **4**, and **5** were synthesized to evaluate their biological activity.

Compound **1**, also known as sakuranetin, was first isolated from the Japanese cherry tree *Puneus pseudo-cerrasus* (Asahina, 1908) in the early 20^th^ century, while compound 2 was first identified as a constituent of South-American *Tessaria dodoniefolia* in 1973 (Kavka et al., 1973). Most recently, 1 and 2 have been reported as constituents of *Siolnatria brasilensis* (dos Santos et al., 2019), *Rhus retinorrhoea* (Mossa et al., 1996) and *Arteminia halodendron* (Sun et al., 2019).

## Materials and Methods

### Plant Material

The leaves and branches of *C*. *thyrsiflora* were collected during the flowering stage from Cuesta Zapata, Melipilla province, Santiago (33° 24’ 01.4’’ S, 71° 15’ 20.7’’ O) in December 2015. The species was recognized by forestry engineer Patricio Novoa Quezada of the National Forestry Corporation. A voucher specimen (001016) is in deposit at the Herbarium of Universidad Técnica Federico Santa María, Valparaiso, Chile.

### Extraction and Isolation of Flavanones

The aerial parts of *C*. *thyrsiflora* were dried at room temperature under protection of sun light for two days. The 3.1 kg of dry and ground plant material was successively extracted on an Edwards and Ceruti stainless steel macerator with two liters of hexanes, dichloromethane, and ethanol for 72 h. The solvents were evaporated under reduced pressure to leave viscous residues.

A sample of 15 g of the dichloromethane extract was column chromatographed on silica gel 60 eluted with hexanes/ethyl acetate (1:1). HF-254 silica gel TLC plates were used to monitor the separation, detecting spots under UV light and by heating after drenching with 10% H_2_SO_4_ in H_2_O. The fractions obtained with hexanes/ethyl acetate 13:7 and 3:2 were separately crystallized using hexanes/ethyl acetate with high temperature.

### Chemical Assays

#### Estimation of the Total Flavonoid Content

The total flavonoid content was determined using the Dowd method as described (Arvouet-Grand et al., 1994), with modifications to adapt the screen of 96-well plates. Briefly, 150 µl of 2% AlCl_3_ in ethanol was mixed with the same volume of extract solution in ethanol (1.0 mg/ml). Absorption readings at 415 nm were taken after 10 min against a blank sample consisting of a 150 µl extract solution with 150 µl methanol without AlCl_3_. The total flavonoid content was determined using a standard curve with quercetin (0–70 mg/L) as the standard (Y = 71.545 x - 0.6373, R^2^ = 0.9994). The mean of three readings was used and expressed as mg of quercetin equivalents (mg QE) per g of extract.

#### Estimation of the Total Phenolic Content

A modified Folin-Ciocalteu (FC) method (Madrid et al., 2015) was used. The sample solution (15 *μ*L) was mixed with distilled water (15 *μ*L) and 0.2 N FC reagent (150 *μ*L). After 5 min, the 3% sodium carbonate solution (120 *μ*L) was added. A mixture of distilled water (15 *μ*L), methanolic sample solution (15 *μ*L), HCl 0.2 N (150 *μ*L), and 3% sodium carbonate solution (120 *μ*L) was used as blanks. The absorbance was measured at 765 nm. The mean of three readings was used and expressed as gallic acid equivalents (GAE; mg/g extract) using a gallic acid (0–500 mg/L) standard curve.

### Statistical Analysis

All data were expressed as the mean ± standard deviation (SD). Graphpad Prism 7 software was used to ascertain statistically significant differences. The differences among multiple groups were evaluated using the one-way analysis of variance non-parametric (Kruskal-Wallis test) with Dunn’s post-test. The difference between the means was considered statistically significant at P < 0.05.

### Spectroscopic Analysis

IR spectra were obtained from KBr pellets on a Thermo Scientific Nicolet 6700 FT-IR spectrophtometer (Waltham, MA, United States), MS analysis were measured on a Thermo Fisher Scientific Exactive™ Plus Orbitrap high-resolution mass spectrometer (Bremen, Germany) at a resolution of 140,000 amu (10% valley), and NMR spectra were measured on a Bruker AC-400 spectrometer (Rheinstetten, Germany) at 400 MHz for ^1^H and 100 MHz for ^13^C in CDCl_3_ for **1** and in DMSO-*d*_6_ for **2**, Spectra were referenced using the residual solvent peaks at *δ* 7.26 (CDCl_3_) and *δ* 2.50 (DMSO) for ^1^H and at *δ* 77.0 and 39.5 for ^13^C, respectively.

### Stereochemical Analyses

#### HPLC-CD-OR Analysis

Electronic circular dichroism and optical rotation analyses were made using a Shimadzu HPLC chromatograph coupled to a JASCO CD-2095plus CD detector and a PDR advanced laser polarimeter detector, and data were acquired using JASCO ChromNAV software version 2.01.05. Chiroptical measurements were obtained through a chromatographic analysis using a Phenomenex Lux Cellulose-1 column (150 mm x 4.5 mm, 5 *µ*m) with a mobile phase composed of *n*-hexane/isopropanol 9:1 at a flow rate of 1.0 ml/min. Optical rotation response was measured at 670 nm, while UV and ECD spectra were measured between 220 and 420 nm by stop-flow while each compound was in the CD detector cell. Although this HPLC-CD-OR measurement methodology was not used for the quantitative analyses of the pure isolated flavanones, a validation procedure was performed using pure racemic flavanone and obtaining consistent results with those described in a previous work (Muñoz et al., 2013).

#### VCD Measurements

IR and VCD spectra were measured on a BioTools Chiral*IR* FT spectrophotometer equipped with dual photoelastic modulation. A sample of 4.3 mg of derivative of 1 with 4’-*O*-acetyl (1AC) in 150 *μ*L of 100% atom-D CDCl_3_ was placed in a BaF_2_ cell with a path length of 100 *μ*m, data were acquired at a resolution of 4 cm^-1^ during 6 h, and the samples stability was monitored by 300 MHz ^1^H NMR spectra immediately prior to and after the VCD measurements.

#### DFT Calculations

A systematic conformational search for 1AC was performed using molecular mechanics as implemented in the Spartan’04 software package (Wavefunction-Inc., 2004). The two conformers obtained when considering a 10 kcal/mol energy cutoff above the global minimum energy value, were submitted to geometry optimization with the Gaussian 03W software package (Frisch et al., 2003) using the B3LYP and B3PW91 hybrid functionals and the DGDZVP basis set. The IR and VCD frequencies obtained at the same level of theory for both low energy conformations were weighted according to a Boltzmann distribution and scaled using an anharmonicity factor of 0.972, which was estimated using the Compare*VOA* software (Debie et al., 2011), and plotted as Lorentzian bands with half-widths of 6 cm^-1^.

### Synthesis of Flavanone Analogues 3, 4, and 5

#### Chemicals

All purchased chemical reagents (Merck or Sigma-Aldrich) were of the highest available purity and used without previous purification. Melting points (mp in °C) were measured on a Stuart-Scientific SMP3 melting point apparatus and are uncorrected.

#### General Procedure for the Protection of Phenolic Compounds With MOMCl

All protected phenolic compound were synthesized as described (Hyun et al., 2010) with some modifications. To a stirred solution of phenolic compound (1.0 equiv.) in CH_2_Cl_2_ (0.12 M concentration) were added i-Pr_2_NEt (1 equiv. per OH group) and MOMCl (1 equiv. per OH group) dropwise at 0°C, and the reaction mixture stirred at room temperature until the reaction completed. The ^1^H, ^13^C-NMR and 2D NMR data were consistent with the reported structures (Khupse and Erhardt, 2007; Du et al., 2017; Barbuščáková et al., 2018).

#### General Procedure for the Synthesis of Chalcones

They were synthesized as described (Ndoile and Heerden, 2013) with some modifications. The reaction mixture was warmed to 50°C and stirred until completion of reaction as monitored by TLC. After work-up the mixtures were recrystallized from ethanol to give the chalcones as pure solids. Their melting point and spectral data are given below.

#### General Procedure for the Synthesis of Flavanones 3, 4, and 5

They were synthesized as described with some modifications (Ketabforoosh et al., 2014). To a stirred solution of chalcone (1 equiv.) in EtOH (5 ml/mmol) were added NaOAc (7 equiv.) and H_2_O (vol. equiv. of EtOH). The reaction mixtures were heated under reflux for 6 h. and then allowed cooling to room temperature. After work-up the flavanone mixtures were purified by column chromatography using hexanes-ethyl acetate mixtures of increasing polarity (Ketabforoosh et al., 2014). The OMOM groups were hydrolyzed with 1N HCl in methanol under reflux for 15 min longer hydrolysis times lowered yield.

### Biological Assays

#### Fungal Strains and Culture Conditions

A strain of *Botrytis cinerea* isolated from a naturally infected grape (*Vitis vinifera*) was used in all experiments. Isolated *Phytophtora cinnamomi* was provided by the Agricultural and Livestock Service of Valparaíso. Both strains were maintained on a potato dextrose agar (PDA) medium (DIFCO, Detroit, MI) at 4°C. The inoculum of both pathogens was grown on PDA and incubates in dark at 23°C for 5 days in the case of *B. cinerea* and 8 days for *P. cinnamomi*.

#### *In* *Vitro* Fungicidal Activity Assays Against *B*. *cinerea* and *P*. *cinnamomi*

The fungicidal activity of flavanones, extracts, the negative control (C-) composed of solvent (1% ethanol), and the positive control (C+, commercial fungicide Captan^®^ and metalaxil^®^ for *B. cinerea* and *P. cinnamomi*, respectively) were assessed using the radial growth test technique on PDA medium as described (Taborga et al., 2015). All test samples were dissolved in ethanol (1%) and water, to obtain final concentrations of 50, 150, and 250 mg/L in the PDA medium. A plug (4 mm) of PDA medium with five-day-old mycelium colonies of the pathogen was placed at the center of a Petri dish with PDA medium with or without studied test samples and were incubated at 23°C in dark for 72 h (*B*. *cinerea*) and 120 h (*P. cinnamomi*). After incubation, the diameters of colonies were measured and the inhibition percentage was calculated as the average of three replicas compared to C-.

#### *In Vitro* Antibacterial Activity Assays, Phytopathogens

The minimum inhibitory concentrations (MIC) were determined using the broth microdilution method (Díaz et al., 2018). Two-fold serial dilutions were added to the wells of sterile 96-well plates containing inoculated BBL™ Mueller Hinton II Broth medium suspensions of bacterial cells (1.0 x10^5^ UFC/ml) of *Agrobacterium tumefaciens* and *Bacillus subtilis*. The final concentrations ranged from 12.5 to 1,600 *µ*g/ml. Ethanol (1%) was used as the negative control and chloramphenicol (CALBIOCHEM; U.S. and Canada) was used as the positive control against both bacteria. MICs were determined according to the OD_600_ obtained in a Thermo Scientific Multiskan GO 96-well plate photometer. After the cultures were incubated at 37°C for 24 h, MIC was confirmed as the lowest concentration that completely inhibited the bacteria growth. Assays were done in triplicate.

#### *In Vitro* Antibacterial Activity Assays, Human Pathogens

Two clinical isolates of methicillin resistant *S*. *Aureus* (622-4 and 97-7), and one clinical isolate of *E*. *coli* 33.1, kindly donated by Dr. Marcela Wilkens from Universidad de Santiago de Chile were used, and *S. aureus* (NCTC8325-4) and *E. coli* (ATCC25922) were used as the control strains. The antimicrobial activities of the isolated compounds against *E. coli* (ATCC25922), *E. coli* multi-resistant (33.1), *S. aureus* (NCTC8325-4), and methicillin-resistant *S. aureus* 97-7 strains, were determined using the microdilution method established by CLSI (NCCLS, 1999). Briefly, stock solutions (5 mg/ml) of compounds in DMSO were diluted in Mueller-Hinton broth (MHB) to the different two-fold assay concentrations. The final concentration of DMSO was ≤ 2.5% and does not affect the microbial growth. The obtained solution was added to MHB and serially two-fold diluted in a 96-well microplate. A sample of 100 *μ*L of inoculum 1.5 × 10^6^ colony forming unit (CFU) ml^-1^ in MHB was added and then incubated at 37°C for 18 h. The assay was repeated three times. Wells containing MHB, 100 *μ*L of inoculum, and DMSO served as negative controls. The MIC was defined as the lowest concentration of compounds resulting in the complete inhibition of visible growth (Felix et al., 2009).

#### *In Vitro* Cytotoxic Activity of Flavanone 2

The used cell lines include MDA-MB-231 human breast adenocarcinoma cells, B16-F10 mouse metastatic melanoma cells and MEF primary mouse embryonic fibroblast. Cells were maintained in a DMEM high glucose medium (Mediatech, Manassas, VA, USA) supplemented with 10% (MDA-MB-231 and B16-F10) or 15% (MEF) heat-inactivated fetal bovine serum (HyClone Laboratories, South Logan, USA), 100 IU ml^-1^ penicillin and 100 *μ*g ml^-1^ streptomycin while maintained at 37°C in a 5% CO_2_ humidified atmosphere. Cell viability was measured using a CyQuant^®^ Direct Cell Proliferation Assay Kit (Life Technologies, Grand Island, NY, USA) following the manufacturer’s instruction. Briefly, 5,000 cells per well were seeded onto a flat-bottom 96-well plate in 200 *μ*L final volume. Six hours after seeding, the culture medium was replaced by the medium containing the tested compounds at 0.01 to 100 *µ*M concentrations in DMSO (0.1% final concentration) during 72 h. Untreated cells (medium containing only 0.1% DMSO) were used as controls. At the end of the incubation, 100 *μ*L of culture medium was removed from each well and replaced by detection reagent solution provided by the kit (which is a 1:5 mixture of the reagent CyQUANT^®^, direct nucleic acid stain and the reagent labeled as CyQUANT^®^ direct background suppressor I). Cells were incubated for 1 h and fluorescence emission was measured at 535 nm with excitation at 480 nm in a microplate reader (Infinite 200 PRO, Tecan, Grödig, Austria). At least four independent experiments were performed for each concentration. The results from each experiment were transformed into percentage of controls and the LC_50_ values were graphically obtained from the dose-response curves. The LC_50_ value was obtained adjusting the dose-response curve to sigmoidal curves (variable slope) using the GraphPad Prisma 6.0 software (GraphPad-Software, 2015).

#### *In Vitro* 5-hLOX and 15-sLOX Enzyme Assays

The commercially available enzyme 5-hLOX from Cayman Chemicals Inc. (Ann Arbor, MI, USA), was diluted (1:500) in the assay buffer. This contained 4-(2-hydroxyethyl)-1-piperazineethane sulfonic acid (HEPES) 50 mM, ethylenediamine tetraacetic acid (EDTA) 2 mM, adenosine triphosphate (ATP) 10 *μ*M, and CaCl_2_ 10 *μ*M at pH 7.5) admixed with 10 *μ*M of H_2_DCFDA. The reaction mixture was incubated for 15 min in the assay plate. Later, 280 *μ*L of buffer and 10 *μ*M of inhibitors were added per well and incubated for 30 min at 37°C. Finally, the reaction was started by adding a suitable concentration of arachidonic acid (0.5 *μ*M) and the fluorescence was read in a multimode detector Synergy™ HT Multi-Mode Microplate Reader (Biotek, Bad Friedrichshall, Germany) at 480 nm exciting at 520 nm after incubation at room temperature for 1 h. The IC_50_ values were obtained for analysis of oxidation of H_2_DCFDA dye to the highly fluorescent 2’,7’-dichloro-fluorescein (DCF) using the non-linear curve-fitting program. The kinetic assays to determine the mechanism of inhibition were done with the best inhibitors, using different concentrations of the inhibitor (0–1 *μ*M) and substrate (0.2–5 *μ*M). All data were collected in duplicate and the assays were performed on different days to ensure reproducibility.

The activity of 15-sLOX (Cayman Chemical Item No. 60712) was determined following the formation of reaction products at 234 nm (ϵ = 25,000 M^-1^ cm^-1^) in a Perkin-Elmer Lambda 25 UV/Vis spectrophotometer. All reactions were performed at a final volume of 2 ml and were constantly stirred using a magnetic bar at room temperature. The reaction medium used contained 25 mM HEPES buffer (pH 7.4), Triton X-100 0.01%, and the arachidonic acid (AA) substrate at a 10 *µ*M concentration determine preliminary percent inhibition (%I). AA concentration was determined quantitatively by allowing the enzymatic reaction to go to completion. The reaction was carried out by adding the inhibitor dissolved in DMSO (5 *µ*g/ml in the reaction medium) to the bucket with the substrate buffer, and finally the enzyme was added. Assays were performed in duplicate on three different days. GraphPad Prism Demo V.7 performed the graphics for IC_50_ and kinetics studies (GraphPad-Software-Inc., 2017).

#### Docking of Flavanone 2 With 5-hLOX

The structure of 2 was built with the Molecular Operating Environment software (Chemical-Computing-Group-ULC, 2018). ChelpG charges were obtained at the B3LYP/6-31G(d,p) level of theory, employing the Gaussian 09 software (Frisch et al., 2016). Docking of all inhibitors into the active site of the crystal structure of 5-hLOX (PDB code: 3O8Y, 2.39 Å resolution) was performed with the AutoDock4 package (Morris et al., 2009) using a Lamarckian algorithm and assuming total flexibility of the inhibitors, the docking validation was done with known inhibitors of 5-hLOX because doesn’t exist co-crystallized inhibitors with the enzyme. The grid maps were made up to 60 × 60 × 60 points, with a grid-point spacing of 0.375 Å and the Fe3^+^ as the center of the grid map. The AutoTors option was used to define the ligand torsions, and the docking results were then analyzed by a ranked cluster analysis, resulting in conformations with the highest overall binding energy (most negative Gibbs free energy binding value, –ΔG).

## Results and Discussion

### Chemical Activity

#### Extracts, Total Flavonoids, Total Phenolics Content, and Compounds Isolated From *C*. *thyrsiflora*

The hexanes, dichloromethane, and ethanol extracts yielded 0.57, 2.5, and 5.2% residues, respectively. Total flavonoids and total phenolic content compounds were measured. The results are shown in **Table 1**. The polar extracts ED and EE had significantly higher total flavonoid and total phenolic compound values than the less polar EH extract. ED extract had a total flavonoid value higher than EE (62.02 ± 0.15 and 46 ± 1.53 mg eq. quercetin/g extract, respectively). For this reason, ED was subjected to column chromatography separation and crystallization of the fractions, flavanones 1 and 2, as white crystals, were obtained in 4.59 and 1.08% yields, respectively. These flavonoid aglycones 1 and 2 were isolated from ED extract, which is expected for this solvent (Grotewold, 2006).

**Table 1:** Total flavonoids and phenolics content of *C*. *thyrsiflora* extracts.

	Extract EH	Extract ED	Extract EE
**Total flavonoids (mg eq. quercetin/g extract ± SD)**	11.51 ± 0.21^a^	62.02 ±0.15^b^	46.77 ± 1.53^c^
**Total phenolic content compounds (mg eq. gallic acid/g extract ± SD)**	18.28 ± 1.68	141.73 ± 3.04	150.40 ± 11.92

^a^p< 0,05 EH versus ED; ^b^p< 0,05 ED versus EE; ^c^p< 0,05 EH versus EE (Kruskal-Wallis test, Dunns post-test for multiple comparison).

The absolute configuration determination of 1 and 2 was ignored in a previously study (Ley et al., 2005) since no optical rotation was accounted.

Previous characterization of the secondary metabolites of *C*. *thyrsiflora* focused on the hexanes, dichloromethane, and ethyl acetate extracts demonstrating the presence of diterpenes (Chamy et al., 1991) and glycosides (Di Fabio et al., 1995). Aromatic compounds and alkaloids have also been extracted from this species and evaluated with their respective antimicrobial activity (Bravo et al., 2005).

### Spectroscopic Characterization of Isolated Compounds

The isolated compounds correspond were identified as (-)-(2*S*)-5,4’-dihydroxy-7-methoxyflavanone (1) and (-)-(2*S*)-5,3’,4’-trihydroxy-7-methoxyflavanone (**2**).

(-)-(2*S*)-5,4’-Dihydroxy-7-methoxyflavanone (1). HRMS *m*/*z*, observed: 285.0769; C_16_H_14_O_5_ [M - H]^-^ requires: 285.0768. IR (KBr) V_max_ cm^-1^: 3529, 3457, 3147, 1630, 1618, 1570. ^1^H NMR (CDCl_3_, 400 MHz) δ: 12.0 (s, 1H, OH-5); 7.34 (d, 2H, *J* = 8.5 Hz, H-2’, H-6’); 6.89 (d, 2H, *J* = 8.6 Hz, H-3’, H-5’); 6.07 (d, 1H, *J* = 2.3 Hz, H-6); 6.04 (d, 1H, *J* = 2.3 Hz, H-8); 5.36 (dd, 1H, *J* = 3.0, 13.0 Hz, H-2); 3.81 (s, 3H, OCH_3_); 3.09 (dd, 1H, *J* = 13.0, 17.0 Hz, H-3b); 2.78 (dd, 1H, *J* = 3.0, 17.0 Hz, H-3a). ^13^C NMR (CDCl_3_, 100 MHz) δ: 196.0 (C-4); 168.0 (C-7); 164.1 (C-5); 162.9 (C-9); 156.1 (C-4’); 130.5 (C-1’); 128.0 (CH-2’, 6’); 115.7 (CH-3’, 5’); 103.1 (C-10); 95.1 (CH-6); 94.2 (CH-8); 79.0 (CH-2); 55.7 (OCH_3_); 43.2 (CH_2_-3). The spectra data were consistent with the reported structure (Vasconcelos et al., 1998).

(-)-(2*S*)-5,3’,4’-Trihydroxy-7-methoxyflavanone (2). HRMS *m*/*z*, observed: 301.0721; C_16_H_14_O_6_ [M - H]^-^ requires: 301.0718. IR (KBr) V_max_ cm^-1^: 3370, 3183, 1638, 1603, 1572. ^1^H NMR (DMSO, 400 MHz) δ: 12.1 (s, 1H, OH-5); 9.14 (s, 2H, OH-3’, OH-4’); 6.92 (s, 1H, H-2’); 6.78 (s, 2H, H-5’, H-6’); 6.11 (dd, 2H, *J* = 2.0, 6.7 Hz, H-6, H-8); 5.45 (dd, 1H, *J* = 2.8, 13 Hz, H-2); 3.82 (s, 3H, OCH_3_); 3.27 (dd, 1H, *J* = 13.0, 17.0 Hz, H-3b); 2.75 (dd, 1H, *J* = 2.8, 17.0 Hz, H-3a). ^13^C NMR (DMSO, 100 MHz) δ: 197.0 (C-4); 167.5 (C-7); 163.3 (C-5); 162.9 (C-9); 145.8 (C-3’); 145.3 (C-4’); 129.4 (C-1’); 118.1 (CH-6’); 115.4 (CH-5’); 114.4 (CH-2’); 102.7 (C-10); 94.7 (CH-6); 93.9 (CH-8); 78.7 (CH-2); 56.0 (OCH_3_); 42.2 (CH_2_-3). MS *m*/*z*: 299.0566, 301.0721, 302.0753. The spectra data were consistent with the reported structure (Mossa et al., 1996).

### Absolute Configuration of 1 and 2 Through Chiroptical Spectroscopy and DFT Calculations

**Figure 1:**
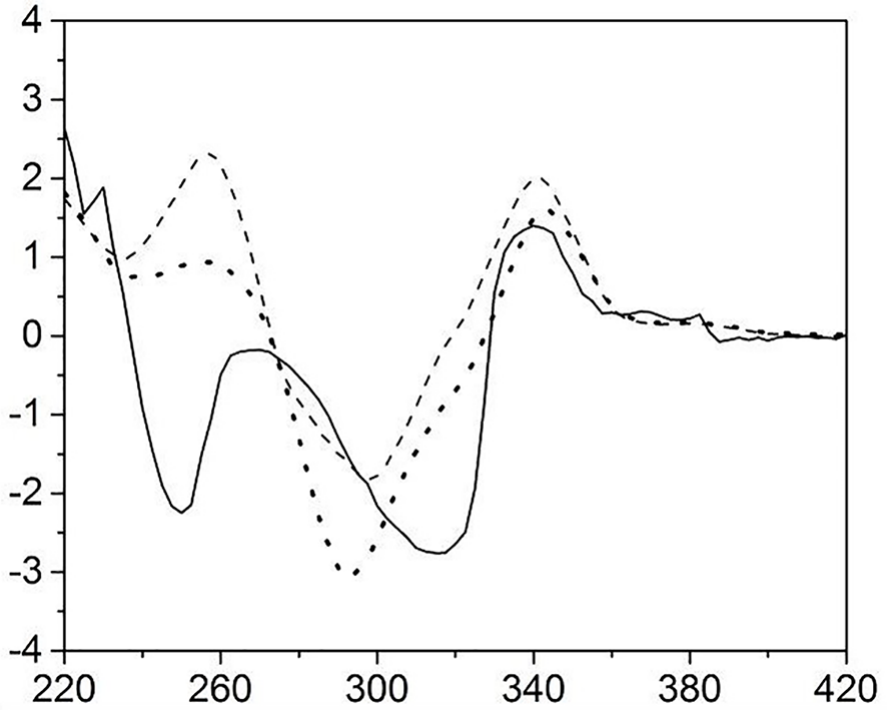
Comparison of the ECD spectra of (-)-(*S*)-1 (dashed line), (-)-(*S*)-2 (dotted line), and (-)-(*S*)-flavanone (continuous line).

Compounds **1** and **2** share the common C-2 stereogenic center and can exist as two enantiomers. As a first step to assign the absolute configuration (AC) of both compounds, optical rotation (OR) and electronic circular dichroism (ECD) measurements were performed using suitable chromatographic detectors coupled to a high-performance liquid chromatograph (HPLC) in a serial arrangement (HPLC-ECD-OR). These measurements showed that both compounds are levorotatory and exhibit nearly identical ECD spectra in the 220–420 nm spectra region (**Figure 1**). These observations clearly suggest that both flavanones possess the same AC as they differ only in the presence of an additional hydroxy group at C-3´ in 2. A literature search reveals that (-)-1 has been associated to the (*S*) enantiomer (Jerz et al., 2005), which suggests both compounds have this AC. The above assumption is further corroborated when ECD traces of both natural products are compared with that of parent (-)-(*S*)-flavanone for which also a very similar spectrum is observed (**Figure 1**).

**Figure 2:**
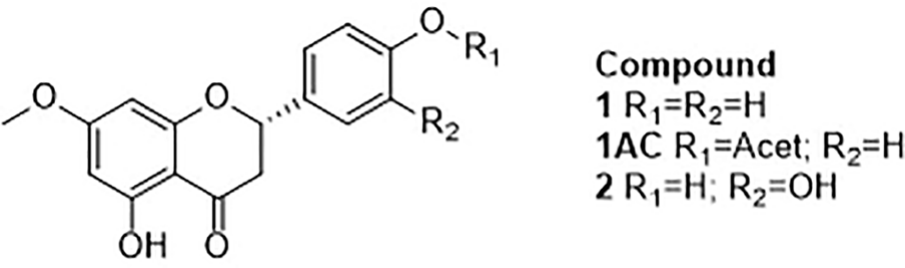
Compounds studied by ECD.

**Figure 3:**
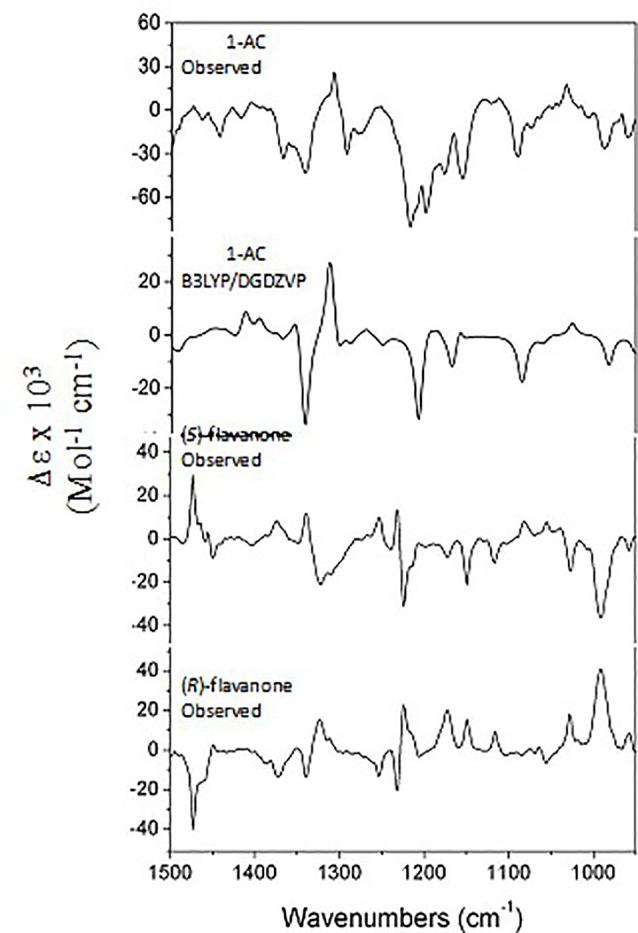
Comparison of the observed VCD spectrum of (-)-(*S*)-1AC with the B3LYP/DGDZVP calculated spectrum for the (*S*) enantiomer, and the observed spectra of (*S*)- and (*R*)-flavanone. The calculated spectra include an anharmonicity factor of 0.972.

Although ECD is widely accepted to establish the absolute configuration of the sole stereogenic center of flavanones, the current study provided the first opportunity to explore vibrational circular dichroism (VCD) spectroscopy for such a task using natural occurring flavanones of known absolute configuration. ECD generally provides a very limited number of informative transitions in the UV region, while VCD provides many bands in the IR region, and therefore VCD is a significantly more robust methodology than ECD. In addition, VCD is not dependent on the use of model compounds as is implicit in ECD studies of flavanones. VCD has shown to be a reliable technique for the assignment of the AC of secondary metabolites (Burgueño-Tapia and Joseph-Nathan, 2015; Joseph-Nathan and Gordillo-Román, 2015) including flavanones (Muñoz et al., 2013). Nevertheless, such studies typically require a 5–10 mg sample to be dissolved in 150 *µl* of CDCl_3_ or CCl_4_. In the cases of 1 and 2, such requirements are not meet due to the poor solubility of either sample in these solvents, and therefore derivatives of polar OH groups to generate acetyl or methoxy containing molecules can overcome such inconveniences thereby allowing successful measurements. In the present case, only 1 was available in the required amounts and thus its treatment with acetic anhydride provided the 4’-*O*-acetyl derivative 1AC (**Figure 2**). Accordingly, the VCD and IR spectra of the later were measured and compared *in silico* with calculated spectra for the (*S*) enantiomer (**Figure 3**). These calculations required a conformational search using the MMX molecular mechanics force field, which afforded only two low energy conformations differing in the C-6–C-7–O–CH_3_ dihedral angle. Both conformers were subjected to geometry optimizations and vibrational calculations using density functional theory (DFT) at the B3LYP/DGDZVP level of theory. The comparisons shows with a 100% confidence level (Debie et al., 2011), that 1AC has the (*S*) AC.

Visual comparison of the VCD spectrum of 1AC with those of (*R*)- and (*S*)-flavanone reported by us (Muñoz et al., 2013), unexpectedly showed a better agreement with the former than with the later enantiomer. This odd observation induced us to revise the VCD spectra of the enantiomers of parent flavanone, which revealed the published spectra (Muñoz et al., 2013) are wrongly labeled. The correct spectra of the two flavanone enantiomers are those presented herein, which when compared with that of **1AC** (**Figure 3**), clearly shows agreement with the (*S*) enantiomer, as expected, and therefore confirms that 1 and 2 share the (*S*) AC as deduced using the OR and ECD observations.

### Synthesis of Flavanones Analogs 3, 4, and 5

**Figure 4:**
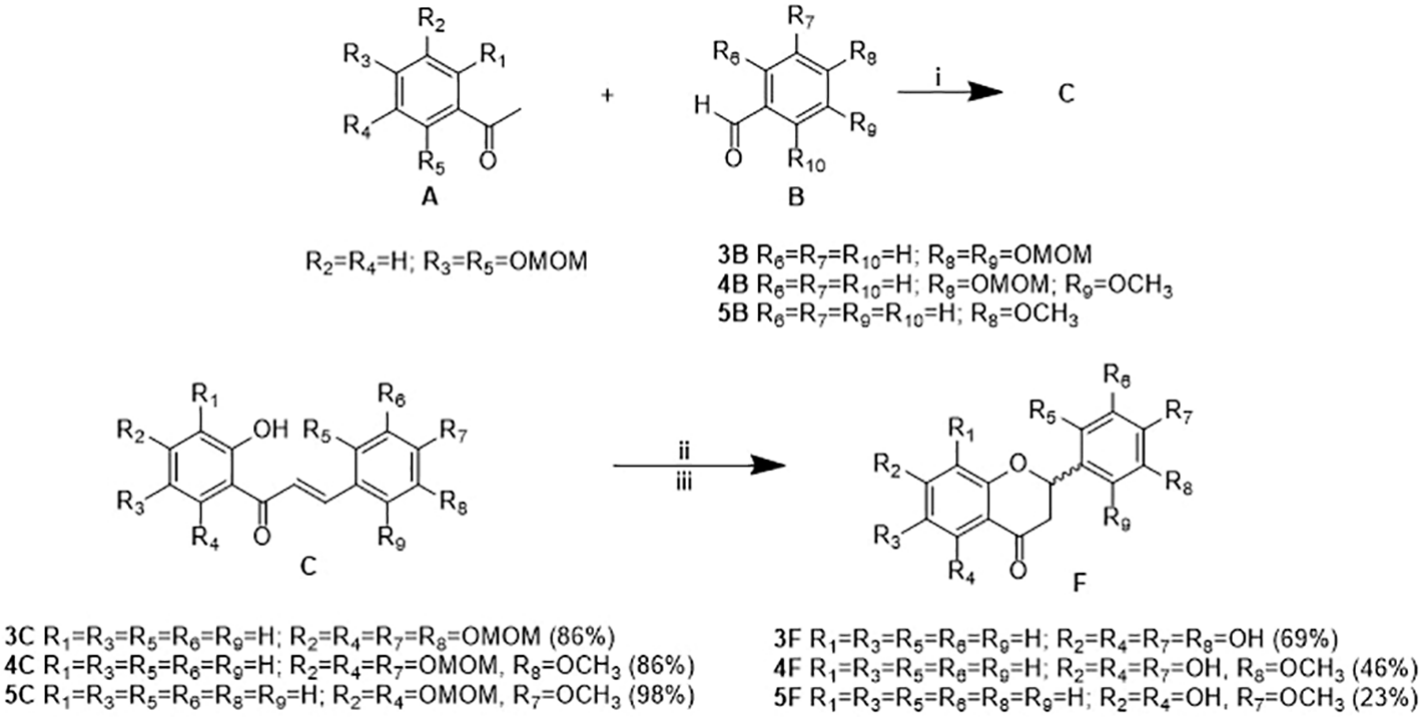
Preparation of flavanones (3, 4, and 5) *via* Claisen-Schmidt condensation in alkaline medium of acetophenone A and benzaldehydes B (i: KOH/MeOH/H_2_O) to produce chalcones C, cyclization of chalcones C under mild alkaline conditions (ii: NaOAc, EtOH-H_2_O, reflux), and acid hydrolysis (iii: HCl 1N, MeOH, reflux) to obtain flavanones F. Reaction yields are shown in brackets.

The synthesis of analogs of the isolated compounds was done to determine the influence of the hydroxy and methoxy groups positions on the biological activity. Thus, Claisen-Schmidt condensation, cyclization under mild alkaline conditions, and acidic hydrolysis as shown in **Figure 4** was undertaken. The reaction of the acetophenone with different benzaldehydes (3B, 4B, and 5B) in alkaline media produced chalcones (3C, 4C, and 5C) in excellent yields (86–98%). Further cyclization and hydrolysis reactions formed flavanones in regular to good yields (23–69%). However, longest hydrolysis times gave poorer yields.

### Spectroscopic Characterization of Chalcones

The synthesized chalcones are 3C, 4C, and 5C.

3-(3,4-bis(Methoxymethoxy)phenyl)-1-(2-hydroxy-4,6-bis(methoxymethoxy)phenyl)prop-2-en-1-one (3C) was obtained as a yellow solid, m.p. 93 - 94°C as reported (Detsi et al., 2009); 1-(2-hydroxy-4,6-bis(methoxymethoxy)phenyl)-3-(3-methoxy-4-(methoxymethoxy)phenyl)prop-2-en-1-one (4C) was obtained as a yellow solid, m.p. 83 - 84°C as reported (Duan et al., 2006); and 1-(2-hydroxy-4,6-bis(methoxymethoxy)phenyl)-3-(4-methoxyphenyl)prop-2-en-1-one (5C) was obtained as a yellow solid, m.p. 97–98°C as reported (Alam and Islam, 2003).

### Spectroscopic Characterization of Flavanones

The synthesized flavanones are 3, 4, and 5.

5,7,3’,4’-Tetrahydroxyflavanone [(±)-eriodictyol] (3) was obtained as white solid (0.86 g, 69%); mp: 265,3 – 267,1°C as reported (Wurm and Geres, 1982); 3’-methoxy-4’,5,7-trihydroxyflavanone [(±)-homoeriodictyol] (4) was obtained as a white solid (0.64 g, 46%); mp: 225,6 - 227,3°C as reported (Zhao et al., 2011); 5,7-dihydroxyl-4’-methoxyflavanone [(±)-isosakuranetin] (5) was obtained as a white solid (0.35 g, 23%); mp: 195–195.5°C as reported (Horowitz and Gentili, 1961) for (-)-isosakuranetin, and (Zhao et al., 2011) for [(±)-isosakuranetin]. All NMR spectra were consistent with those reported.

### Biological Assays

#### *In* *Vitro* Fungicidal Activity of Extracts and Isolated Compounds

**Table 2** shows that all concentrations of the dichloromethane extract (ED) have high activity only against *P*. *cinnamomi*, while only hexanes extracts (EH) exhibit inhibitory activity against *B*. *cinerea*. None of these extracts showed significant activity compare to Captan and Metalaxil. In turn, only the ED extract showed activity against *P*. *cinnamomi*; although neither 1 nor 2 explain the activity of the extract. Due to the low activity of the extract, the compounds were not analyzed against *B*. *cinerea*. There are no reported studies of antifungal activity of extracts or compounds isolated from *C*. *thyrsiflora*.

**Table 2:** Antifungal activity of extracts and compounds isolated from *C. thyrsiflora* on *in vitro* mycelial growth of *B*. *cinerea* and *P*. *cinnamomi*.

Extract or compound	Percentage of inhibition on *in vitro* mycelial growth of *B. cinerea* (%)			Percentage of inhibition on *in vitro* mycelial growth of *P. cinnamomi* (%)		
	50 mg/L	150 mg/L	250 mg/L	50 mg/L	150 mg/L	250 mg/L
EH	0.0 ± 0.0	62.0 ± 2.9	70.0 ± 5.6	0.0 ± 0.0	0.0 ± 0.0	0.0 ± 0.0
ED	0.0 ± 0.0	19.0 ± 1.0	36.0 ± 0.1	64.0 ± 0.6	86.0 ± 0.9	91.0 ± 0.7
EE	0.0 ± 0.0	0.0 ± 0.0	0.0 ± 0.0	0.0 ± 0.0	0.0 ± 0.0	30.0 ± 9.8
1	N.D	N.D	N.D	0.0 ± 0.0	0.0 ± 0.0	0.0 ± 0.0
2	N.D	N.D	N.D	0.0 ± 0.0	33.0 ± 5.8	42.0 ± 5.3
Captan	94 ± 5.2	100 ± 0.1	100 ± 0.2	–	–	–
Metalaxil	–	–	–	100 ± 0.2	100 ± 0.1	100 ± 0.1

N.D, Not determined.

#### *In* *Vitro* Antibacterial Effects of Pathogenic Bacteria to Plant Extracts and Metabolites

EH and ED showed high activity against both phytopathogenic bacteria, unlike the more polar extract EE (**Table 3**) but lower than chloramphenicol but the isolated compounds have no antimicrobial activity against the studied phytopathogenic bacteria.

**Table 3:** Antibacterial activity of extracts and compounds isolated from *C. thyrsiflora* against pathogenic bacterial strains.

Extract or compound	Minimum inhibitory concentration (MIC) µg/ml	
	GN *A*. *tumefaciens*	GP *B*. *subtilis*
EH	50	50
ED	50	50
EE	200	400
1	N.A	N.A
2	N.A	N.A
Chloramphenicol	12.5	12.5

G.N, Gram-Negative Bacteria; G.P, Gram-Positive Bacteria; N.A, Not activity.

#### *In* *Vitro* Antibacterial Effects of Pathogenic Bacteria to Humans

Testing the antibacterial activity of all isolated and synthesized compounds against the resistant strains *S*. *aureus* NTCC 8325-4, *S*. *aureus* MRSA 97-7, *S*. *aureus* MRSA 622-4, *E*. *coli* ATCC25922, and *E*. *coli* 33.1 (**Table 4**) revealed that only 2 exhibited moderate inhibitory activity against methicillin-resistant strain *S*. *aureus* MRSA 97-7, with a MIC value of 50 [*µ*g/ml]. It is striking that this molecule has certain selectivity towards the multiresistant strain MRSA 97-7, showing a lower action towards the sensitive strain (NTCC8325-4), or towards the other resistant strain MRSA 622-4. None of the molecules showed activity against the Gram-negative strains (*E*. *coli*).

**Table 4:** Antibacterial activity of isolated and synthesized flavanones against resistant strains bacteria.

Compound	Minimum inhibitory concentration (MIC) µg/ml				
	*S. aureus*NTCC8325-4	*S. aureus*MRSA 97-7	*S. aureus*MRSA 622-4	*E.coli* ATCC25922	*E.coli*33.1
1	>100	>100	>100	>100	>100
2	>100	50	>100	>100	>100
3	>100	>100	>100	>100	>100
4	>100	>100	>100	>100	>100
5	>100	>100	>100	>100	>100
Vancomicin	3,5	25	5	–	–
Ampicillin	–	–	–	60	>100

Previous studies evaluated the antibacterial activity of some secondary metabolites extracted from *C*. *thyrsiflora* (Bravo et al., 2005). The tested compounds were 2,4-dihydroxy-2H-1,4-benzoxazin-3(4*H*)-one (DIBOA), 2-benzoxazolinone (BOA), 2-dydroxy-2H-1,4-benzoxazin-3(4*H*)-one (HBOA), 2,7-dihydroxy-2*H*-1,4-benzoxazin-3(4*H*)-one (7-OH-HBOA), and gallic acid, showing inhibitory activity of bacterial growth in high concentrations (1,000 *µ*g/ml). The activity of these compounds disappears at lower concentrations (250 *µ*g/ml), with the exception of gallic acid, which is the only molecule possessing catecholic hydroxyls, achieving 67.2% inhibition activity of *S*. *aureus* ATCC.

#### *In Vitro* Cytotoxic Activity of Flavanones 1 and 2

As in the previous case, **Table 5** shows that 1 showed no significant cytotoxic activity against MDA-MB-231 (human breast adenocarcinoma cell line), B16-F10 (mouse melanoma cells), or MEF (primary mouse embryonic fibroblast), while 2 exhibits a 4-fold higher selectivity towards B16-F10 as compared to the effect on non-cancerous cells (MEF). Flavanone 2 also exhibited cytotoxic activity against MDA-MB-231, but with no selectivity over MEF. When the cytotoxic effect of 2 against B16-F10 was compared with commonly used chemotherapeutic drugs, it showed 4.6- and 17-fold less activity than etoposide and taxol, respectively. These activity have shown to be important for cancer cells proliferation and viability (Ghosh and Myers, 1998; Steinhilber et al., 2010; Chen et al., 2016) and we previously showed that B16-F10 are more sensitive to drugs that interfere with 5-hLOX activity (Vásquez-Martínez et al., 2019).

**Table 5:** Cytotoxic activity of constituents of *C*. *thyrsiflora*.

Compound	LC_50_ (µM) ± SD		
	MDA-MB-231	B16-F10	MEF
1	∼ 1100	72.02 ± 7.16	145.6 ± 23.4
2	48.72 ± 9.32	8.07 ± 1.61	31.01 ± 3.69
Taxol	0.21 ± 0.48	0.48 ± 0.08	0.40 ± 0.07
Etoposide	1.33 ± 0.28	1.74 ± 0.32	1.03 ± 0.17

#### *In Vitro* 5-hLOX and 15-sLOX Enzyme Assays

The enzymatic results (**Table 6**) showed that both flavones were selective inhibitors of 5-hLOX. This difference could be explained comparing the binding sites of LOX since is known that the cavity of 5-hLOX is 20% larger than 15-sLOX (Mitra et al., 2015) being dependent of molecular volume and shape and the possibility to enter into the binding site. The IC_50_ results showed that 2 was a more potent inhibitor (0.10 *µ*M) than 1 (11.31 *µ*M) for 5-hLOX and a structure evaluation confirms that the catechol group is a key factor for the interaction with the enzyme. Flavanone 2 showed the greatest 5-hLOX inhibitor activity which correlated with a higher cytotoxic activity.

**Table 6:** Biological activity of isolated and synthesized flavanones as 5-hLOX and 15-sLOX inhibitors.

Compound	% of inhibition(5-hLOX)	% of inhibition(15-sLOX)	IC_50_(5-hLOX)	*K_i_*(5-hLOX)	Type of inhibition
1	61 ± 1.341	7 ± 3.125	11.31 µM ± 3.022	4.71 µM ± 1.542	non-competitive
2	93 ± 0.791	26 ± 3.955	0.10 µM ± 0.081	0.05 µM ± 0.012	competitive
3	88 ± 0.678	N.D	0.22µM ± 0.023	0.07µM ± 0.013	competitive
4	11.67 ± 2.45	N.D	N.D	N.D	N.D
5	2.57 ± 2.26	N.D	N.D	N.D	N.D
NDGA	93 ± 5.870	N.D	0.33 µM ± 0.15	N.D	competitive

Flavanones at 10 µM; IC_50_, Half inhibitory concentration; Ki, inhibitory constant; N.D, not determined; NDGA, nordihydroguaiaretic acid.

**Figure 5:**
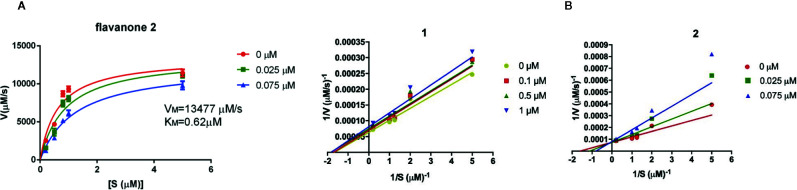
**(A)** Michaelis-Menten for flavanone 2; **(B)** The Lineweaver-Burk graph of the inhibition of 5-hLOX by flavanones 1 and 2.

The above is confirmed with the kinetic results (**Figures 5A, B**), showing that 2 is a competitive inhibitor (0.05 µM) of 5-hLOX, meanwhile 1 presented a non-competitive mechanism (4.71 µM). The potent *in vivo* anti-inflammatory effects of 2 found in several assays related to its neuroprotective action (Fischer et al., 2019), could be in part explained by the inhibition of 5-hLOX found in this work.

#### Docking of Flavanone 2 With 5-hLOX

**Figure 6:**
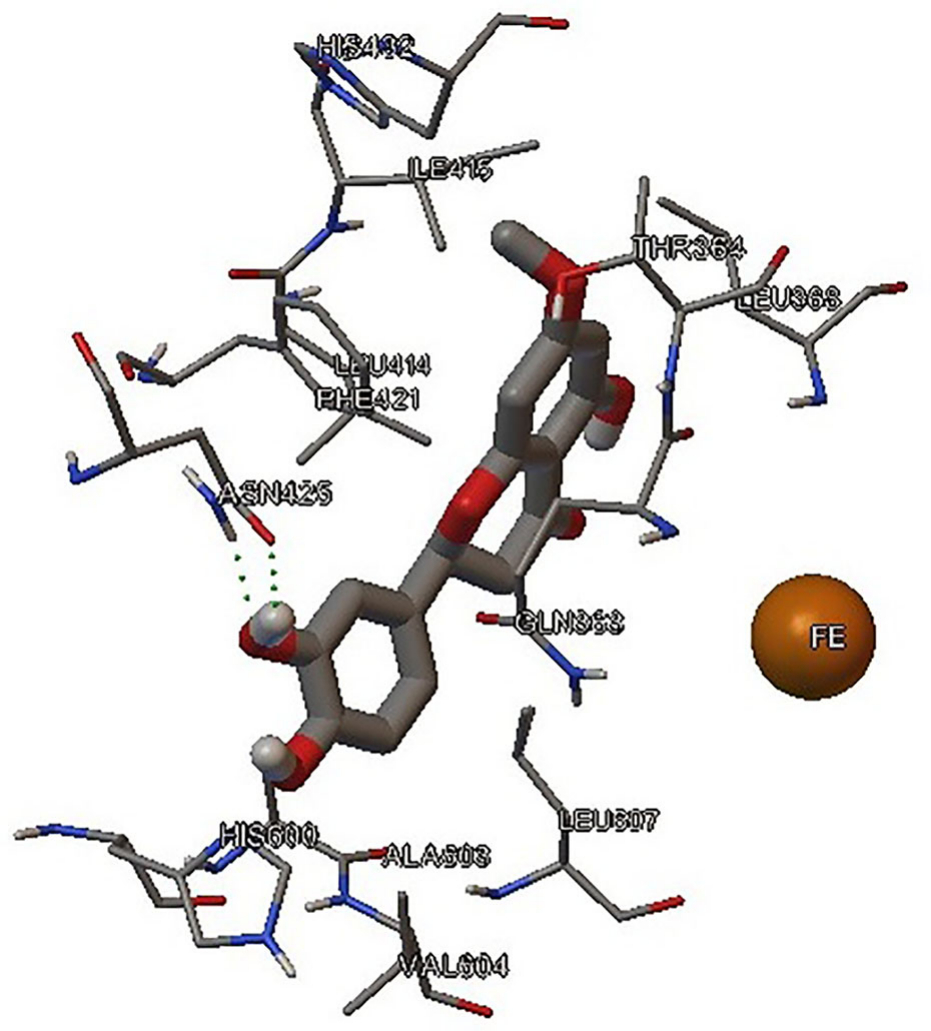
Docking result of flavanone 2 and 5-hLOX.

Docking studies were done for the best 5-hLOX inhibitor. The results (**Figure 6**) showed that (*S*)-2 located inside the binding site very near to the catalytic iron (energy binding -1.53 kcal/mol) in accordance to a competitive mechanism, instead, we observed positive binding energy with the flavanone 1 into the binding site of the enzyme that agrees with the non-competitive mechanism. We also studied the docking of the (*R*)-isomer whose results showed not a good energy binding value (-0.42 kcal/mol). It was noted that the 3’-OH of flavanone 2 forms a hydrogen bounding with ASN425 favoring the binding between 2 and 5-hLOX, also, the structure shows hydrophobic interactions between the aromatics rings of flavanone 2 and the residues as PHE, ILE, and LEU, finally the cation-pi interaction is observed between the flavanone and iron in the catalytic site, the same behavior is observed for the other good inhibitor founded for 5-hLOX that is flavanone 3 that confirm the relevance of the catechol group and the inhibition of the enzyme.

## Conclusions

*In* *vitro* fungicidal activity and antibacterial effects on bacteria pathogenic to plants show that, although the ED has the best activity, it is much lower than the controls used and the isolated compounds are not responsible for this activity. Flavanone **2** has selectivity activity in cytotoxic tests against B16-F10 and in the *in vitro* antibacterial effects on bacteria pathogenic to human against *S*. *aureus* MRSA 97-7. In the *in vitro* 5-hLOX enzyme assays, flavanone 2 again has more activity than **1**, probably due to the presence of catecholic hydroxyls that is an important characteristic founded in 5-hLOX inhibitors. This result validates the use in the traditional medicine of the aerial parts of *C*. *thyrsiflora* as an anti-inflammatory. The Docking study shows that the activity is related to the presence of the 3’-OH group. However, when comparing 2 with 2, that although both compounds possess catecholic hydroxyls, 2 has a higher activity probably due to its stereochemistry. Finally, it should be noted that the (*S*) absolute configuration of 1 and 2 was determined with certainty by ECD and VCD for the first time. We have also verified that flavanone 1 is (*S*)-sakuranetin and 2 is (*S*)-sterubin. The docking study also confirms this stereochemistry.

## Data Availability Statement

The raw data supporting the conclusions of this manuscript will be made available by the authors, without undue reservation, to any qualified researcher.

## Author Contributions

LT and MO designed the investigation. EV carried out the extraction, separation, and purification of compounds. LT and EV recollected the plant material and collaborated on the structure determination of 1 and 2. CG collaborated on the structure determination of 1 and 2. MO carried out the synthesis of 3–5 and evaluated the total flavonoids and total phenolic content. KD performed the *in vitro* anti-phytopathogenic activity assays. MM carried out the OR, ECD, and VCD calculations. PJ-N carried out the VCD measurements and comparisons. YV-M and MC-M performed the *in vitro* antibacterial activity assays against human pathogenic bacteria and statistical analysis of the chemical activity. CM and CT performed the *in vitro* 5-hLOX and 15-sLOX enzyme assays and FC carried out the molecular docking studies with 5-hLOX enzyme and 2. MM and SM carried out the *in vitro* cytotoxic activity assays. All authors contributed to the article and approved the submitted version.

## Funding

This work was supported by FONDECYT (No. 1130924, No. 1141071), PIIC-DGIIP of Universidad Técnica Federico Santa María, DICYT-USACH (No. 021901VM, No. 021941MC) of Universidad de Santiago de Chile, and CONACYT-Mexico (No. 284194).

## Conflict of Interest

The authors declare that the research was conducted in the absence of any commercial or financial relationships that could be construed as a potential conflict of interest.
