# Supplementary material for: Biological Properties and Absolute Configuration of Flavanones From Calceolaria thyrsiflora Graham
Source: Front Pharmacol. 2020 Jul 28;11:1125. doi: 10.3389/fphar.2020.01125 (PMC7399337; doi:10.3389/fphar.2020.01125)
Supplement: Supplementary file 1 [file DataSheet_1.docx]

Supplementary Material

**Supplementary Figure 1.** ^1^H, ^13^C, ^13^C DEPT-135 spectra of compound **1**.

**Supplementary Figure 2.** ^13^C DEPT-90 spectra of compound **1**.

**Supplementary Figure 3.** Gs-2D Heteronuclear Single Quantum Coherence (HSQC) spectra of compound **1**.

**Supplementary Figure 4.** Gs-2D  Heteronuclear Multiple Bond Correlation (HMBC) spectra of compound **1**.

**Supplementary Figure 5.** High resolution mass spectra of compound **1**.

**Supplementary Figure 6.** Fourier transform infrared (FT-IR) spectra of compound **1**.

**Supplementary Figure 7.** ^1^H, ^13^C, ^13^C DEPT-135 spectra of compound **2**.

**Supplementary Figure 8.** Gs-2D Heteronuclear Single Quantum Coherence (HSQC) spectra of compound **2**.

**Supplementary Figure 9.** Gs-2D  Heteronuclear Multiple Bond Correlation (HMBC) spectra of compound **2**.

**Supplementary Figure 10.** High resolution mass spectra of compound **2**.

**Supplementary Figure 11.** Fourier transform infrared (FT-IR) spectra of compound **2**.

**Supplementary Figure 12.** ^1^H spectra of compound **3C**.

**Supplementary Figure 13.** ^13^C spectra of compound **3C**.


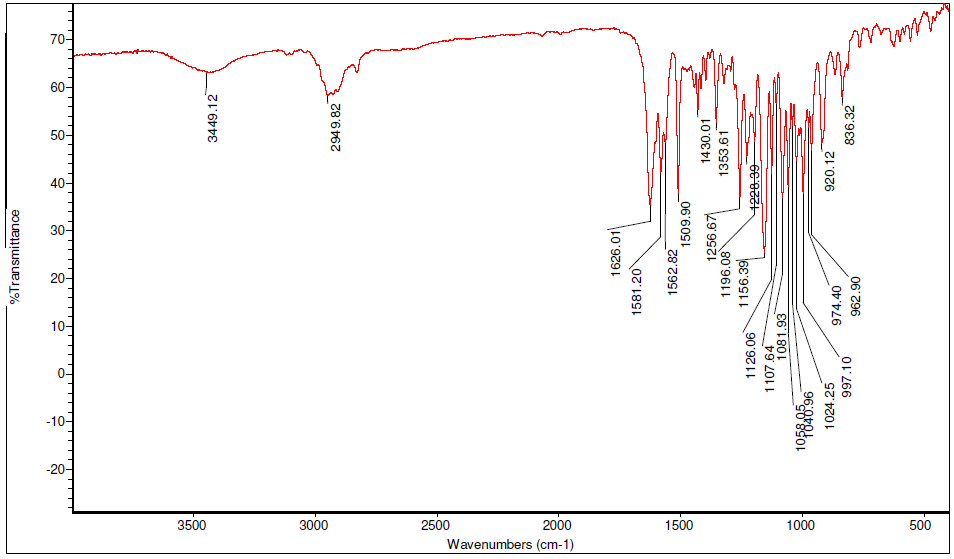


**Supplementary Figure 14.** Fourier transform infrared (FT-IR) spectra of compound **3C**.

**Supplementary Figure 15.** ^1^H spectra of compound **4C**.

**Supplementary Figure 16.** ^13^C spectra of compound **4C**.


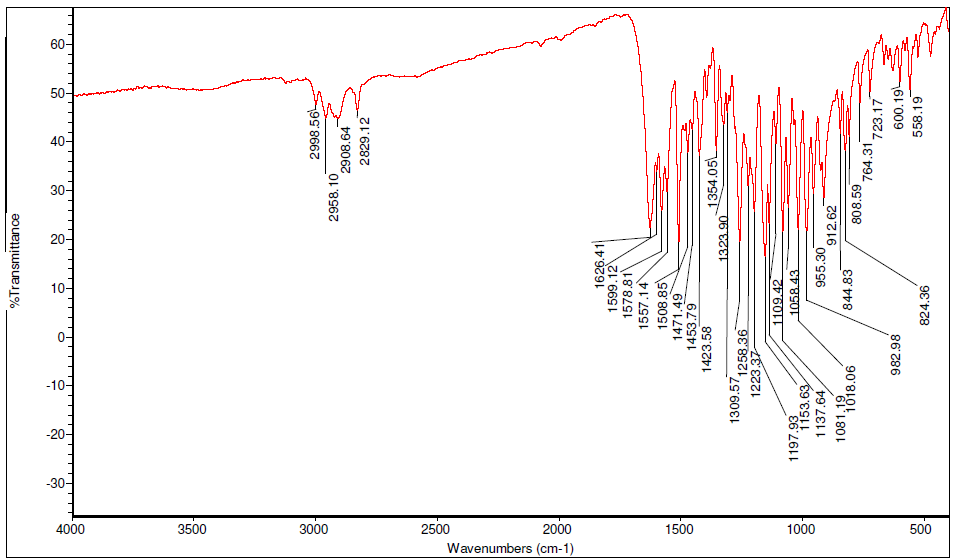


**Supplementary Figure 17.** Fourier transform infrared (FT-IR) spectra of compound **4C**.

**Supplementary Figure 18.** ^1^H spectra of compound **5C**.

**Supplementary Figure 19.** ^13^C spectra of compound **5C**.


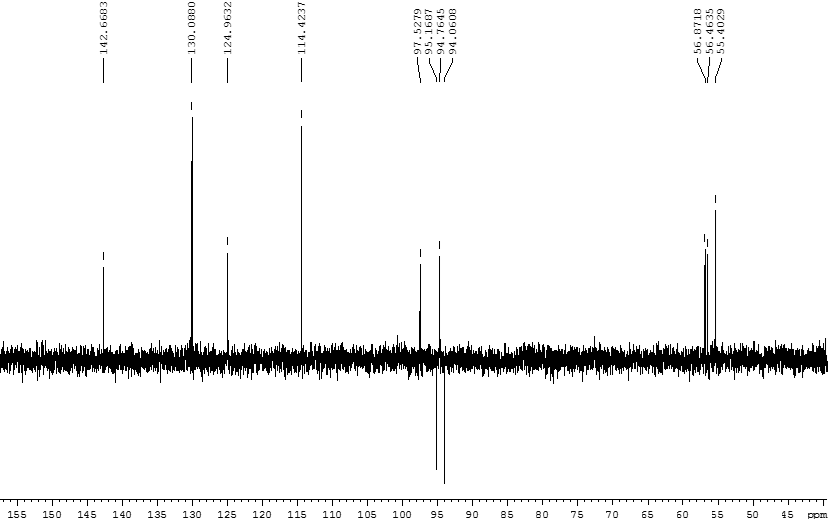


**Supplementary Figure 20.** ^13^C DEPT-135 spectra of compound **5C**.


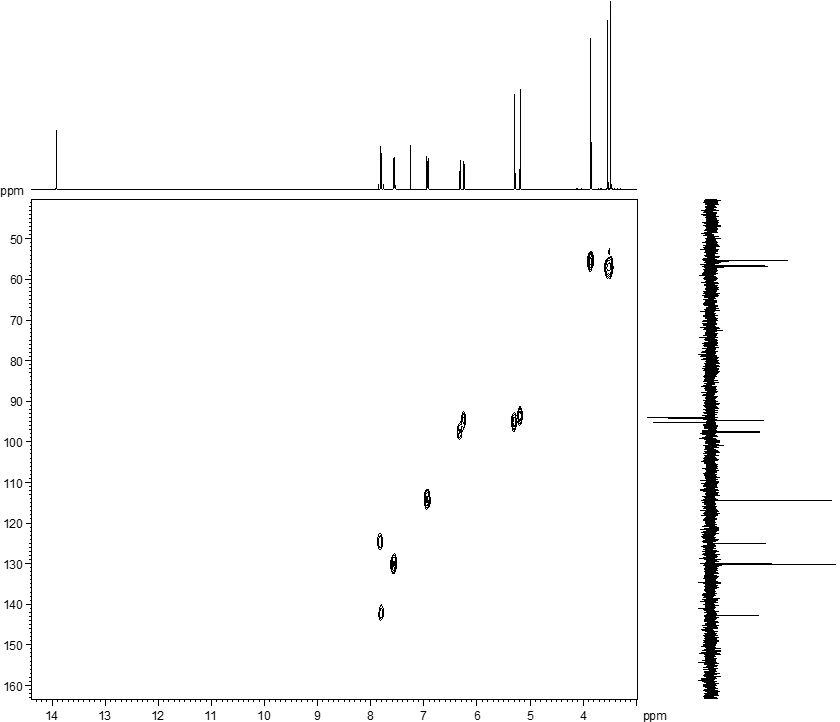


**Supplementary Figure 21.** Gs-2D Heteronuclear Single Quantum Coherence (HSQC) spectra of compound **5C**.


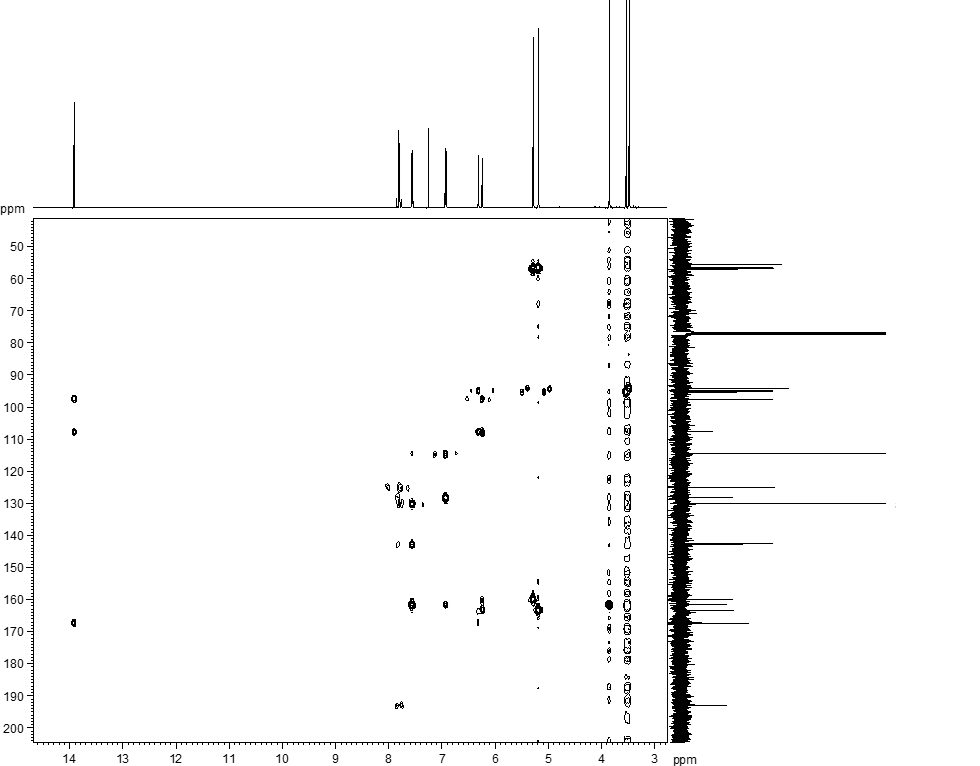


**Supplementary Figure 22.** Gs-2D  Heteronuclear Multiple Bond Correlation (HMBC) spectra of compound **5C**.


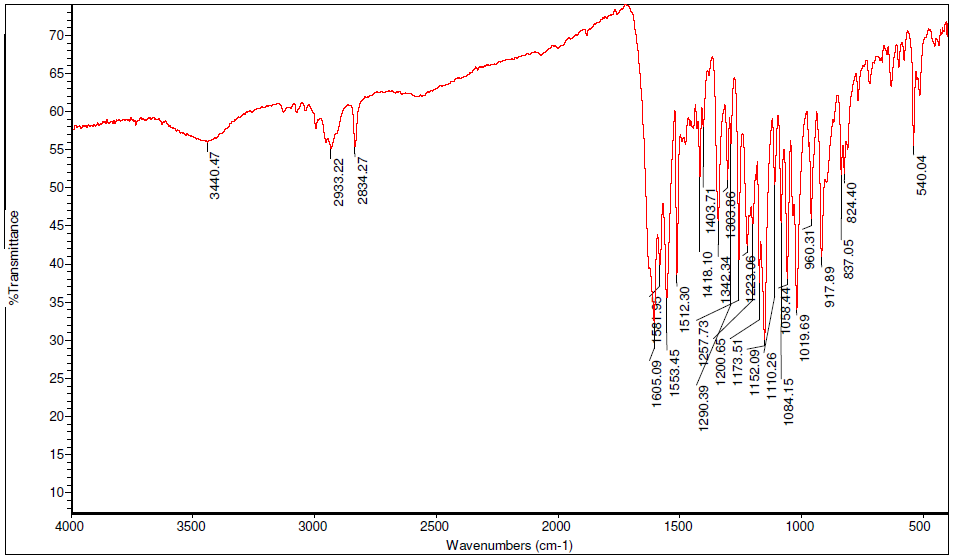


**Supplementary Figure 23.** Fourier transform infrared (FT-IR) spectra of compound **5C**.

**Supplementary Figure 24.** ^1^H spectra of compound **3**.

**Supplementary Figure 25.** ^13^C spectra of compound **3**.

#

**Supplementary Figure 26.** ^1^H spectra of compound **4**.

#

**Supplementary Figure 27.** ^13^C spectra of compound **4**.

**Supplementary Figure 28.** ^1^H spectra of compound **5**.

**Supplementary Figure 29.** ^13^C spectra of compound **5**.


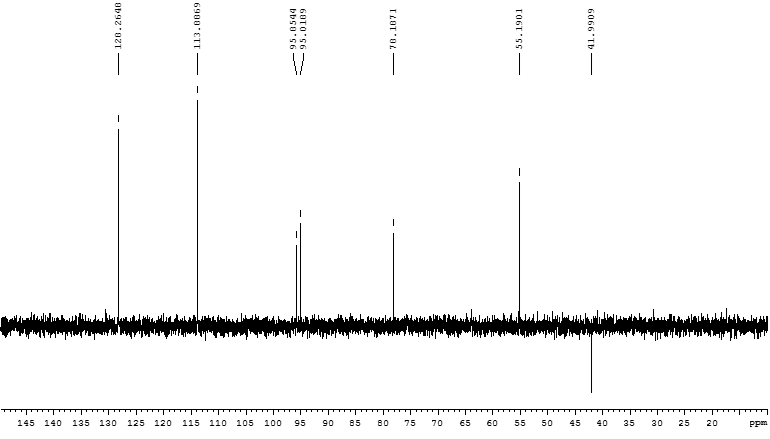


**Supplementary Figure 30.** ^13^C DEPT-135 of compound **5**.


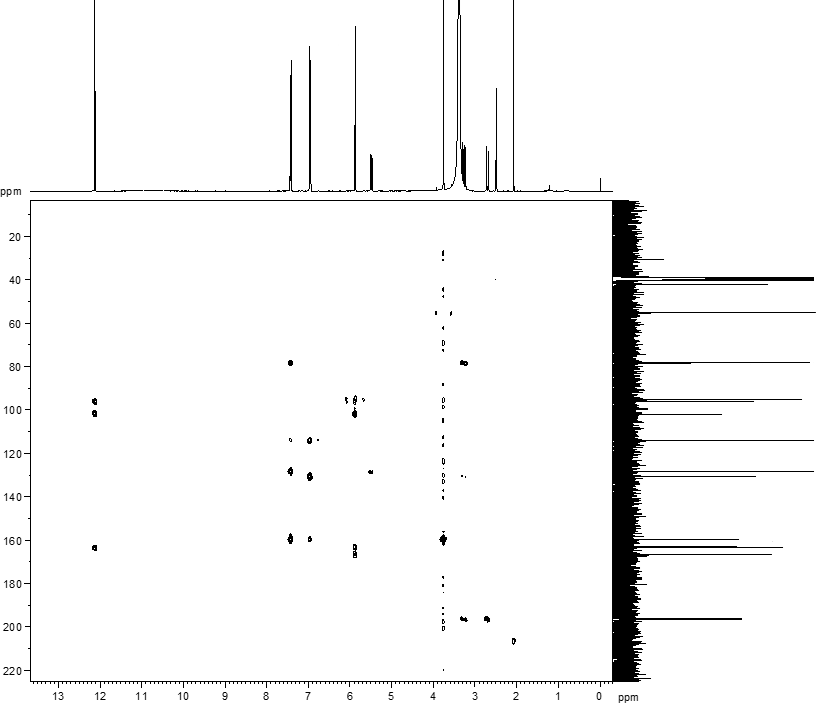


**Supplementary Figure 31.** Gs-2D  Heteronuclear Multiple Bond Correlation (HMBC) spectra of compound **5**.


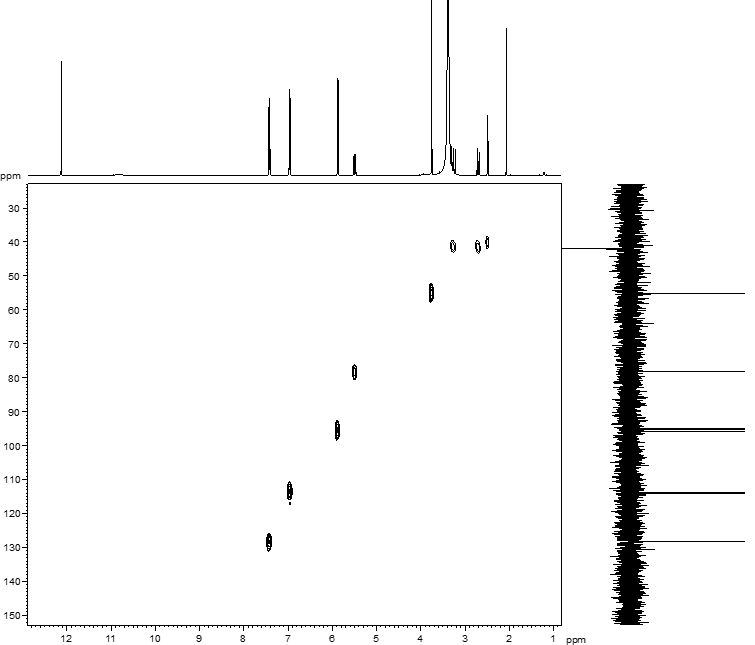


**Supplementary Figure 32.** Gs-2D Heteronuclear Single Quantum Coherence (HSQC) spectra of compound **5**.


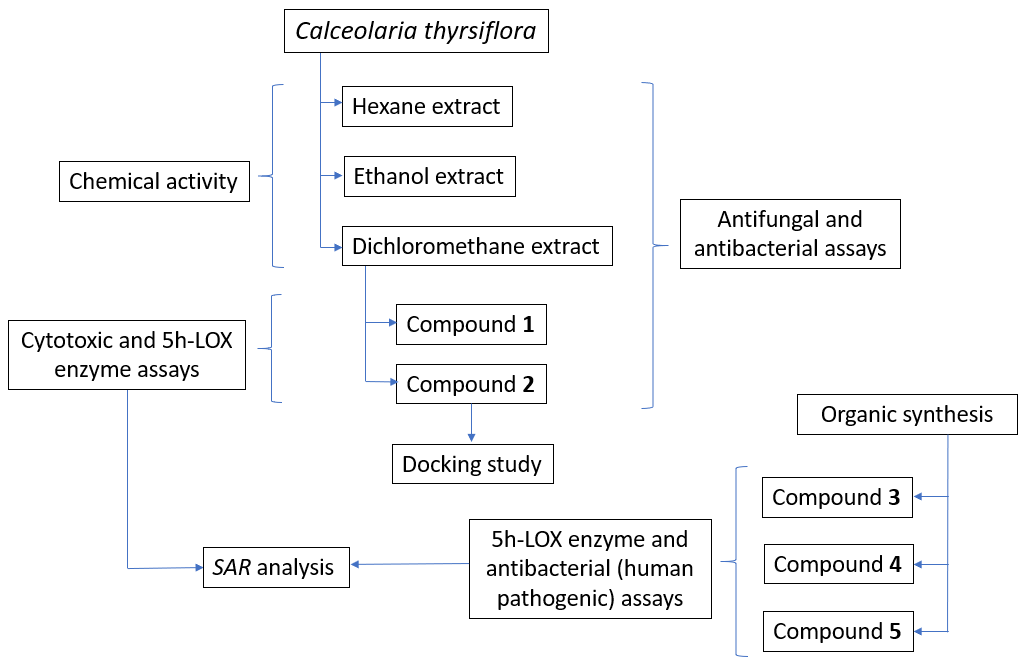


**Supplementary Figure 33. Diagram of the study**.
